# Re-evaluation of protein neutron crystallography with and without X-ray/neutron joint refinement

**DOI:** 10.1107/S2052252522003657

**Published:** 2022-04-08

**Authors:** Takeshi Murakawa, Kazuo Kurihara, Motoyasu Adachi, Katsuhiro Kusaka, Katsuyuki Tanizawa, Toshihide Okajima

**Affiliations:** a Department of Biochemistry, Faculty of Medicine, Osaka Medical and Pharmaceutical University, 2-7 Daigakumachi, Takatsuki, Osaka 569-8686, Japan; b Institute for Quantum Life Science, Quantum Life and Medical Science Directorate, National Institutes for Quantum Science and Technology, 2-4 Shirakata, Tokai, Ibaraki 319-1106, Japan; c Frontier Research Center for Applied Atomic Sciences, Ibaraki University, 162-1 Shirakata, Tokai, Ibaraki 319-1106, Japan; d Institute of Scientific and Industrial Research (SANKEN), Osaka University, 8-1 Mihogaoka, Ibaraki, Osaka 567-0047, Japan

**Keywords:** neutron crystallography, X-ray/neutron joint refinement, copper amine oxidases, quinone cofactor

## Abstract

Protein neutron crystallography is a powerful technique to determine the positions of H atoms, providing crucial biochemical information such as the protonation states of catalytic groups and the geometry of hydrogen bonds. Recently, the crystal structure of a bacterial copper amine oxidase was determined by joint refinement using X-ray and neutron diffraction data sets at resolutions of 1.14 and 1.72 Å, respectively [Murakawa *et al.* (2020 ▸). *Proc. Natl Acad. Sci. USA*, **117**, 10818–10824]. While joint refinement is effective for the determination of the accurate positions of heavy atoms on the basis of the electron density, the structural information on light atoms (hydrogen and deuterium) derived from the neutron diffraction data might be affected by the X-ray data. To unravel the information included in the neutron diffraction data, the structure determination was conducted again using only the neutron diffraction data at 1.72 Å resolution and the results were compared with those obtained in the previous study. Most H and D atoms were identified at essentially the same positions in both the neutron-only and the X-ray/neutron joint refinements. Nevertheless, neutron-only refinement was found to be less effective than joint refinement in providing very accurate heavy-atom coordinates that lead to significant improvement of the neutron scattering length density map, especially for the active-site cofactor. Consequently, it was confirmed that X-ray/neutron joint refinement is crucial for determination of the real chemical structure of the catalytic site of the enzyme.

## Abstract 2

The crystallographic structure of a bacterial copper amine oxidase was determined solely using neutron diffraction data at 1.72 Å resolution. Although the neutron scattering length densities thus obtained were sufficient to determine the locations of all atoms, including H and D atoms, it was confirmed that joint refinement with high-resolution X-ray diffraction data provided more accurate structural features that were not visible in the structure refined from the neutron data alone.

## 1. Introduction

The experimental determination of H-atom positions in biological macromolecules such as proteins provides important biochemical information, including the deprotonation or protonation states of dissociable groups and hydrogen-bond networks, and allows a better understanding of their underlying molecular mechanism. Neutron protein crystallography is a widely known diffraction method for directly locating H atoms in a protein structure, but X-ray crystallography, a conventional diffraction method for structural determination, is unable to do so unless an extremely high resolution is achieved (Ogata *et al.*, 2015 ▸; Hirano *et al.*, 2016 ▸; Liebschner *et al.*, 2020 ▸). While X-ray radiation can result in the reduction of transition metals and redox cofactors or the modification of reactive groups through highly reactive species such as hydroxyl and hydrogen radicals in protein crystals, neutron crystallography determines structures that are essentially unaffected by such radiation damage. Despite its damage-free features, as the neutron beam is generated by nuclear fission at nuclear reactors or by nuclear spallation at accelerators (Schröder & Meilleur, 2020 ▸), the neutron beam flux is relatively weak in contrast to the X-ray beam flux at synchrotron facilities. Therefore, even if diffraction measurements are performed over a long time (more than ten days) using an extra-large crystal (several mm^3^), it is not easy to obtain high-resolution neutron diffraction data. In addition, because the number of H (D) atoms is typically comparable to that of heavy atoms in a protein structure, neutron crystallography needs to determine a larger number of unknown parameters (atom coordinates, *B* factors and/or occupancies based on the refinement strategy) than are required for X-ray crystallography at the same resolution (Niimura & Podiarny, 2011 ▸). To overcome this weakness of neutron crystallo­graphy, an X-ray diffraction data set is usually collected from the same crystal and is combined with that from neutron diffraction for structural refinement. The joint X-ray and neutron refinement (joint refinement) method was first applied in protein crystallography in 1982 (Wlodawer & Hendrickson, 1982 ▸). Since then, this refinement method has been improved by the introduction of many algorithms and functions, especially for refinement of the positions, occupancies and *B* factors of H and D atoms (Afonine *et al.*, 2010 ▸). Joint refinement tends to avoid the local minima that may occur when a medium-resolution structure is refined solely using neutron structure-factor amplitudes. Joint refinement has now become a standard method for neutron crystallography, and the refinement programs for the joint method built into *Phenix* (Liebschner *et al.*, 2019 ▸) and *nCNS* (a patch program for *CNSsolve*; Brünger *et al.*, 1998 ▸) are routinely used for neutron crystallography of proteins.

Copper amine oxidases (CAOs) occur widely in biological species ranging from bacteria to plants and animals, and catalyse the oxidative deamination of various primary amines to produce the corresponding aldehydes, hydrogen peroxide and ammonia (McIntire & Hartmann, 1993 ▸; Klema & Wilmot, 2012 ▸). The CAO family commonly has a homodimeric subunit structure with a prosthetic Cu^2+^ ion and a protein-derived quinone cofactor, topaquinone (2,4,5-trihydroxyphenylalanine quinone; TPQ), in the active site of each subunit. The organic redox cofactor TPQ is post-translationally synthesized from a specific tyrosine residue encoded in the polypeptide chain of all CAOs (Matsuzaki *et al.*, 1994 ▸; Klinman & Mu, 1994 ▸; Kim *et al.*, 2002 ▸; Okajima & Tanizawa, 2009 ▸). After single-turnover biogenesis of TPQ, the mature enzyme can then catalyse the multi-turnover amine-oxidation reaction (Kishishita *et al.*, 2003 ▸; Chiu *et al.*, 2006 ▸; Murakawa *et al.*, 2020 ▸). Recently, we succeeded in obtaining a neutron diffraction data set from a CAO crystal derived from *Arthrobacter globiformis* (AGAO) at a resolution of 1.72 Å, which is unprecedentedly high for a protein containing over 400 residues. Together with X-ray diffraction data from the same crystal at a resolution of 1.14 Å, the crystal structure of the oxidized form of AGAO (PDB entry 6l9c) was determined by the joint refinement method (Murakawa *et al.*, 2020 ▸). However, a question has been raised as to whether the structure obtained from the neutron scattering length density (SLD) map determined only from the neutron diffraction data is identical to that obtained from the joint refinement of neutron and X-ray diffraction data. Ideally, unique structural features, including the positions of H and D atoms, should be preserved in the structure solved only using neutron diffraction data, although some ambiguity may remain since the neutron diffraction data set had a lower resolution. In addition, it is considered that unique structural information may be lost through refinement when combining the X-ray diffraction data, which could potentially include undesirable changes resulting from X-ray irradiation as described above. It has been reported that structural change is probably induced by X-ray irradiation even in joint refinement (Unno *et al.*, 2015 ▸). Comparative studies have previously been conducted between structures obtained using only neutron diffraction data and structures determined by joint refinement (Adams *et al.*, 2009 ▸). In these studies, the SLD maps obtained from the neutron data alone showed less fit to the model than those obtained from joint refinement. Thus, some SLD maps of the H/D atoms cannot be seen in the neutron analysis alone (Blakeley *et al.*, 2008 ▸; Yamaguchi *et al.*, 2009 ▸). This difference might result from the fact that the neutron diffraction data did not have a high resolution or that the refinement programs at the time were less developed than current programs.

In this letter, we describe the structure of AGAO solved only from neutron diffraction data at a resolution of 1.72 Å and compare it with the previously published structure determined by joint refinement. Moreover, we performed joint refinement using moderate-resolution X-ray data (1.72 Å) to evaluate the significance of using high-resolution X-ray diffraction data.

## 2. Methods and methods

### 2.1. Neutron and X-ray diffraction experiments

The neutron diffraction data set at a resolution of 1.72 Å obtained in the previous study (Murakawa *et al.*, 2020 ▸) was also used in this study. Briefly, the extra-large AGAO crystal (about 7 mm^3^) used for diffraction measurements was thoroughly deuterated in a cryo D_2_O solution (3.0 *M* deuterated malonate solution pD 7.4) and was flash-cooled in a cryostream at 100 K. The measurements were obtained under cryogenic conditions with the time-of-flight method for data collection using the Ibaraki Biological Crystal Diffractometer (iBIX) installed on BL03 at the Materials and Life Science Experimental Facility (MLF) of the Japan Proton Accelerator Research Complex (J-PARC), Tokai, Japan. The images obtained were processed with the improved data-processing software *STARGazer* (Yano *et al.*, 2018 ▸).

An X-ray diffraction data set was selected from four data sets that we collected at 100 K on BL5A at the Photon Factory (PF), Tsukuba, Japan from the same crystal as used for the neutron diffraction measurements by changing the radiation point. The selected data set was at approximately 1.7 Å resolution and was distinct from that used in the previous study. Data were integrated, merged and scaled to 1.72 Å resolution using *XDS* (Kabsch, 2010 ▸). For convenience, the analyses using both X-ray data at 1.14 Å resolution and neutron diffraction data (Murakawa *et al.*, 2020 ▸), both X-ray data at 1.72 Å resolution and neutron diffraction data, and only neutron diffraction data are termed ‘X-ray/neutron analysis’, ‘X-ray (1.72 Å)/neutron analysis’ and ‘neutron analysis’, respectively.

### 2.2. Structure refinement

To determine the structure of AGAO by neutron analysis, molecular replacement was performed with *Phaser* version 1.19 (McCoy *et al.*, 2007 ▸) for the data at 1.72 Å resolution. The atomic coordinates of the AGAO monomer (PDB entry 3wa2; Murakawa *et al.*, 2013 ▸) were used as a search model after removing all water molecules. The initial model obtained was refined using *Phenix* (Liebschner *et al.*, 2019 ▸). Manual rebuilding was carried out using *Coot* (Emsley *et al.*, 2010 ▸), and water molecules were added stepwise to the model during refinement. The protonation (deuteronation) states of amino-acid residues and the orientations of water molecules were manually adjusted by carefully examining the SLD map using *Coot*. The positions and *B* factors of all H/D atoms were refined using *Phenix*, together with occupancy refinement. For joint refinement using the X-ray and neutron diffraction data both at 1.72 Å resolution, molecular replacement and pre­liminary structural refinement were first performed using only the X-ray data. Finally, refinement was performed with both sets of diffraction data using *Phenix*.

The model of TPQ was constructed based on the *F*_o_ − *F*_c_ omit map. In the X-ray/neutron analysis (Murakawa *et al.*, 2020 ▸), because the omit map corresponding to the TPQ plane was apparently curved, we relaxed the geometry restraints of TPQ in the Crystallographic Information File (CIF) to fit the model to the electron density (ED). On the other hand, during the model construction of TPQ in the neutron and X-ray (1.72 Å)/neutron analyses, we applied the standard CIF, in which TPQ is geometrically restrained to have a flat structure, to the omit maps.

The Ramachandran plot was calculated using *MolProbity* (Chen *et al.*, 2010 ▸) for structure validation. The data-collection and refinement statistics are summarized in Table 1 ▸. *PyMOL* version 2.1 (Schrödinger) was used to draw figures. Atomic coordinates and structure-factor amplitudes obtained from the neutron analysis and those from the X-ray (1.72 Å)/neutron analysis were deposited in the Protein Data Bank (http://www.rcsb.org/) with accession codes 7wno and 7wnp, respectively, as shown in Table 1 ▸.

## 3. Results and discussion

### 3.1. Data quality and completeness

**Figure 1:**
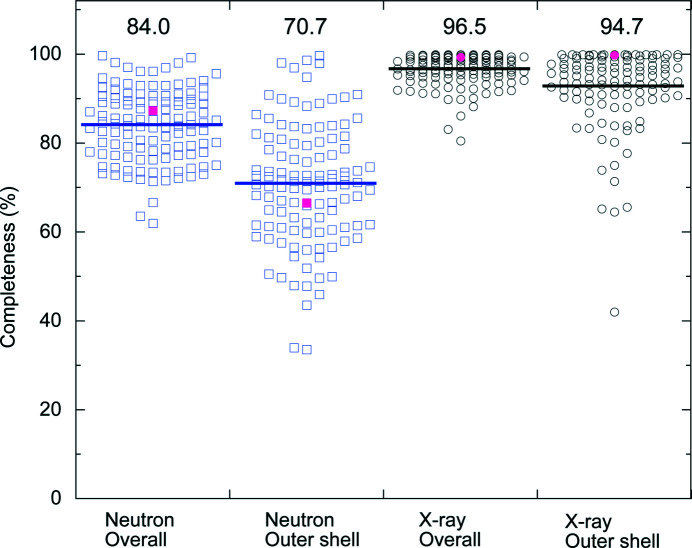
Distribution of the completeness values of neutron and X-ray diffraction data sets in joint-refined PDB data. Completeness values for the overall data and the outer shell were separately plotted for neutron (blue squares) and X-ray diffraction (black circles) data sets in PDB data (*n* = 117) that were obtained by joint refinement. An average value for each group is indicated by solid lines and the numbers shown above (%). The corresponding values in our previous study (PDB entry 6l9c; Murakawa *et al.*, 2020 ▸) are shown as magenta squares and circles.

As described above, the relatively weak neutron beam flux often provides rather low-completeness diffraction data. Indeed, the overall and outer-shell completeness values of neutron diffraction data sets, including those of AGAO, were found to be significantly lower than those of X-ray diffraction data sets in deposited PDB data determined by joint refinement (Fig. 1 ▸). The low completeness of the neutron diffraction data for AGAO (Table 1 ▸) is probably due to the insufficient number of data sets (a total of 33 sets using a wavelength of 3.0–5.7 Å with a detector distance of 491.1 mm) for measuring this enzyme crystal in a low-symmetry space group (*C*2). Nonetheless, the Wilson plot maintains rough linearity to a resolution of 1.72 Å (Supplementary Fig. S1). It is also noteworthy that the lower resolution limit of the neutron diffraction data (20.94 Å) was higher than that of the X-ray diffraction data (50 Å) because of the differences in the detector arrangement between iBIX equipped with multiple neutron detectors surrounding the crystal (minimum 2θ angle 15.5°; Nakajima *et al.*, 2017 ▸) and BL5A equipped with a Quantum 315r CCD X-ray detector (ADSC, California, USA) with a small beam stop. Consequently, we used the data in the range 20.94–1.72 Å for structure refinement.

The subunit of AGAO is the largest among neutron crystal structures reported to date (Supplementary Fig. S2). It is the only structure that contains more than 400 residues with a resolution far exceeding 2.0 Å. Such high-resolution neutron crystallography was achieved by using an extra-large AGAO crystal made by a modified hanging method. Notably, the neutrons generated at MLF operated with a high proton beam power (500 kW) and the highly sensitive detectors of the neutron diffractometer under cryogenic conditions (100 K; Kusaka *et al.*, 2013 ▸) contributed significantly to the measurement of high-resolution diffraction data. Furthermore, the improved *STARGazer* data-processing software for the time-of-flight method also supported the acquisition of structural data at a remarkably high resolution.

### 3.2. Overall structure

In the previous study (Murakawa *et al.*, 2020 ▸), phase determination was performed by molecular replacement using the coordinates of PDB enty 3wa2 as a search model and preliminary structural refinement was performed using the X-ray diffraction data only; joint refinement was then conducted by adding the neutron diffraction data. In the present work, the neutron diffraction data set was used for molecular replacement, and the final coordinates reported in the previous study (PDB entry 6l9c; Murakawa *et al.*, 2020 ▸) were not used as a search model to avoid bias from the joint-refined structure. Further refinement cycles were also completed using only the neutron diffraction data. Thus, the crystal structure of AGAO was determined independently from the previous high-resolution X-ray diffraction data and the joint-refined coordinates. The statistical values for refinement in the neutron analysis are shown in Table 1 ▸. The difference between the *R*_work_ and *R*_free_ values in the neutron analysis (0.094) was larger than that in the X-ray/neutron analysis (0.028), implying some degree of overfitting, although the *R* factor was reduced (from 0.1865 to 0.1632). The overall structure obtained in the neutron analysis is essentially identical to that solved by X-ray/neutron analysis (the r.m.s.d. of the main-chain atoms is 0.120 Å), indicating that refinement using only the neutron data reaches the same solution as the X-ray/neutron analysis. The differences in the dihedral angles of side chains (N—C^α^—C^β^—C^γ^) between the X-ray/neutron and neutron analyses were within 10° for 91.8% of the amino-acid residues. Thus, even with the distinct diffraction data, the orientation of the side chains is suggested to be essentially identical (Supplementary Fig. S3).

In the comparative study of aldose reductase reported previously (Blakeley *et al.*, 2008 ▸), the *B* factor obtained from refinement using only neutron diffraction data at 2.2 Å resolution was higher than that obtained from joint refinement, resulting in an unclear SLD map. In the present study, probably due to the high-resolution neutron diffraction data, the average protein *B* factor in the neutron analysis (20.37 Å^2^) was lower than that in the X-ray/neutron analysis (24.63 Å^2^) (Table 1 ▸) and the SLD map was clear in the neutron analysis. As an example, SLDs of *F*_o_ − *F*_c_ polder omit maps (Liebschner *et al.*, 2017 ▸) for D atoms of the main and side chains were calculated between Gln306 and Glu312 (Supplementary Fig. S4). In this region, the H atoms attached to the N atoms were completely deuterated, and positive densities in the polder omit map for D atoms are as clear as those in the X-ray/neutron analysis. However, for the side-chain H atoms with motional freedom (such as the hydroxyl group of Ser310), SLDs were detected at slightly different positions from those in the X-ray/neutron analysis. To evaluate the quality of the neutron analysis, it is noteworthy that the water molecules, which were assigned on the basis of ED in the X-ray/neutron analysis, were also assigned with SLD in the neutron analysis, and that both analyses detected a similar number of water molecules. About 64% (719/1115) of the water molecules detected by neutron analysis were found at approximately the same positions (within 1.0 Å) as those obtained by X-ray/neutron analysis. Water molecules inside the protein molecule or that were strongly hydrogen-bonded were detected at nearly the same positions. However, some of the secondary bound water molecules, which are slightly distant from the surface of the protein, were detected in only one of the structures. Moreover, when detecting multiple conformers indicating thermal motion and conformational variability, we detected 55 residues with multiple conformations by X-ray/neutron analysis and 21 residues by neutron analysis (Table 1 ▸). These results suggest that joint refinement using high-resolution X-ray data is also effective as a means of detecting multiple conformers.

### 3.3. Active-site structure

**Figure 2:**
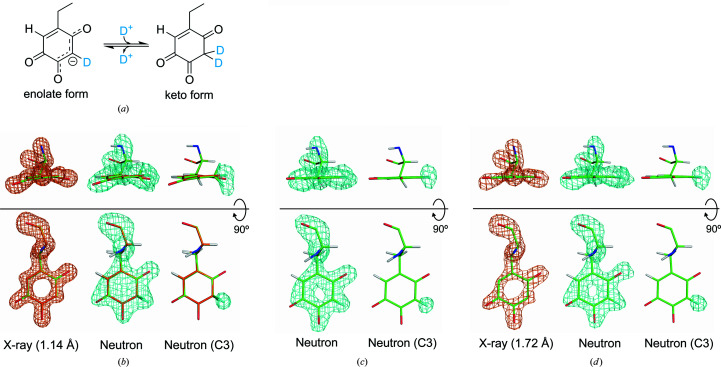
Quinone cofactor structure refined with various data sets. (*a*) Equilibrium between the enolate and the keto forms of TPQ. Refined structure of TPQ from (*b*) X-ray/neutron analysis, (*c*) neutron analysis and (*d*) X-ray (1.72 Å)/neutron analysis. The assigned model of TPQ is superimposed on the *F*_o_ – *F*_c_ polder omit ED map [orange mesh, contoured at 7.0σ for residue 382 (TPQ) in (*b*) and (*d*)] or on the *F*_o_ – *F*_c_ polder omit SLD map [cyan mesh, contoured at 4.0σ for residue 382 and at 3.5σ for D atom(s) attached to the C3 atom of residue 382 in (*b*), (*c*) and (*d*)].

In the active-site structure determined by neutron analysis, the cofactor TPQ and the prosthetic copper ion coordinated by three His residues (His431, His433 and His592) and two water molecules in distorted pyramidal geometry were located at essentially the same positions as those found in the X-ray/neutron analysis. The previous joint-refined structure revealed that TPQ adopts an equilibrium state between the keto and enolate forms, based on the nonplanar model of the quinone ring obtained from the atomic resolution X-ray diffraction data and on the shape of the SLD map at the C3 position of TPQ [Figs. 2 ▸(*a*) and 2 ▸(*b*)]. In the neutron analysis of the present study, we also detected deuteration at the C3 position of TPQ [Fig. 2 ▸(*c*)]. However, based on the shape of the SLD map, a single D atom is assumed to be bound to the C3 position of TPQ, unlike the two atoms assigned in the X-ray/neutron analysis. Moreover, the SLD map in the neutron analysis using the standard CIF file for TPQ fitted better to the planar model of the quinone ring at several sigma levels, presumably due to the lack of high-resolution structure information from X-ray diffraction (Supplementary Fig. S5). Because the position of the C3 atom was not accurately determined in the neutron analysis, it is likely that the SLD corresponding to the D atoms attached to the C3 atom was also affected. Thus, it would be hard to find the existence of the keto form of TPQ (a nonplanar quinone ring and two D atoms attached to the C3 position) from the neutron analysis. This also implies that it is difficult to determine the keto form of TPQ from joint refinement using moderate-resolution X-ray diffraction data. In fact, we performed joint refinement using X-ray and neutron diffraction data both at a resolution of 1.72 Å (Table 1 ▸), but we were unable to unambiguously detect the bent structure with a nonplanar quinone ring from the moderate-resolution ED map [Fig. 1 ▸(*d*)], which gave only a flat TPQ model. These results demonstrate that the non­planar conformation of the TPQ ring is the cofactor structure that can be derived solely from the joint refinement with the high-resolution (1.14 Å) X-ray data (Murakawa *et al.*, 2020 ▸).

**Figure 3:**
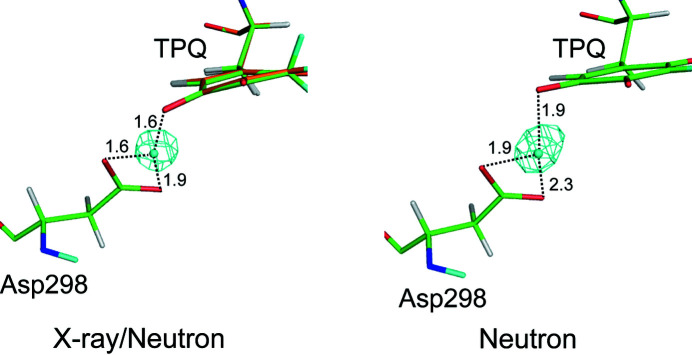
Triply shared proton between the cofactor and the catalytic base refined by X-ray/neutron analysis (left) and neutron analysis (right). *F*_o_ − *F*_c_ polder omit SLD maps, calculated without contributions from the delocalized deuteron, are drawn as a cyan mesh contoured at 4.0σ with respective distances in Å.

It should also be mentioned that we detected SLD, assignable as a deuteron, between the side-chain carboxylate of Asp298 and O5 of TPQ in the neutron analysis (Fig. 3 ▸). However, because the quinone ring is modelled to be flat, the distances between these three O atoms (O^δ1^ and O^δ2^ of the Asp298 side chain and O5 of TPQ) and this shared deuteron in the neutron analysis are longer by 0.3–0.4 Å than those in the X-ray/neutron analysis. Comparison of the structural data revealed that not only the side chain of TPQ, but also the shared proton and the O atoms of the carboxyl group of Asp298, are slightly different in their positions (Supplementary Fig. S6). Advanced quantum-chemical calculations, including nuclear quantum effects, are required to describe various types of proton delocalization due to strong hydrogen bonds (Shoji *et al.*, 2020 ▸). Accurate geometrical information on the shared proton, Asp298 and TPQ is essential for computational analyses in the future, and therefore the importance of the joint refinement is again emphasized.

**Figure 4:**
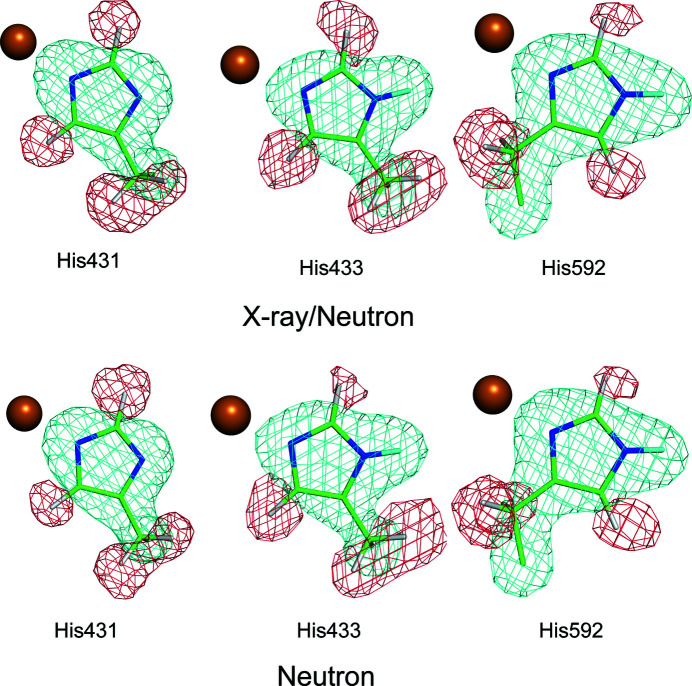
Assigned models of Cu^2+^-coordinated His residues refined by X-ray/neutron analysis (top) and neutron analysis (bottom). *F*_o_ − *F*_c_ polder omit SLD maps, calculated without contributions from His431, His433 and His592, are contoured with positive (cyan) and negative (red) contours of 4.0σ and −3.0σ, respectively. The orange sphere represents the Cu atom.

Moreover, the previous joint-refined structure showed that His431 adopts a fully deprotonated form (an imidazolate anion; Murakawa *et al.*, 2020 ▸). This was the first structural evidence of metal-induced deprotonation (Murakawa *et al.*, 2020 ▸). The present neutron analysis (Fig. 4 ▸) also indicates that His431 is fully deprotonated, with the H^δ1^ atom undetected. These findings reveal that the overall structure and unique features of the active site obtained with the neutron diffraction data only are consistent with those of the X-ray/neutron analysis, albeit with less accuracy.

To summarize, most of the H and D atoms were detected using only neutron diffraction data at a resolution of 1.72 Å, although the SLD map could be further improved by obtaining data with higher completeness. The proton beam power at MLF in J-PARC at the time of this measurement was 500 kW, and the intensity as of 2021 was 740 kW. The beam power is expected to reach 1 MW within a few years. Indeed, the MLF was successfully operated stably (but only for one hour) at a beam power of 1 MW in July 2018 (J-PARC, 2018 ▸). Moreover, the current first target station in J-PARC will be upgraded to a more powerful one with a higher pulse peak (Tanaka *et al.*, 2020 ▸). As the beam intensity increases, the measurement time per data set will decrease, leading to a large number of data sets being obtainable within the same beam time. Therefore, the problem of low completeness will be gradually improved in the near future. Because a neutron beam causes essentially no radiation damage to crystals, the increased beam intensity directly benefits protein crystallo­graphers. However, even if the low completeness is overcome, joint refinement with high-resolution X-ray data provides more accurate heavy-atom coordinates, which in turn could improve the SLD for H/D atoms. Consequently, more accurate protonation states and proton positions are expected to provide the real chemical structure. 

## Supplementary Material

PDB reference: copper amine oxidase from *Arthrobacter globiformis*, neutron data only, 7wno

PDB reference: X-ray (1.72 Å)/neutron analysis, 7wnp

Supplementary figures. DOI: 10.1107/S2052252522003657/hi5655sup1.pdf

## Figures and Tables

**Table 1:** Data-collection and refinement statistics Values in parentheses are for the outer shell.

	X-ray/neutron analysis†		Neutron analysis	X-ray (1.72 Å)/neutron analysis	
	PDB entry 6l9c †		PDB entry 7wno	PDB entry 7wnp	
	X-ray	Neutron	Neutron	X-ray	Neutron
Data collection					
Beamline	BL5A, PF	BL03, J-PARC		BL5A, PF	
Wavelength (Å)	1.0	3.0–5.7		1.0	
Space group	*C*2			*C*2	
*a*, *b*, *c* (Å)	157.55, 61.78, 92.23			157.21, 61.98, 92.45	
α, β, γ (°)	90, 112.13, 90			90, 112.13, 90	
Resolution range (Å)	50.00–1.14 (1.16–1.14)	20.94–1.72 (1.78–1.72)		44.55–1.72 (1.82–1.72)	
*R*_int_	0.069 (0.801)	0.188 (0.596)		0.053 (0.240)	
〈*I*/σ(*I*)〉	46.6 (2.00)	4.99 (1.03)		9.27 (2.54)	
Completeness (%)	99.4 (99.9)	87.4 (66.6)		93.1‡ (91.3)	
Multiplicity	5.2 (3.5)	2.66 (1.62)		1.91‡ (1.90)	
Observed reflections	1526790	203270		308652	
Unique reflections	296297 (14901)	76306 (5748)		161381 (25556)	
Refinement					
Resolution range (Å)	45.03–1.14 (1.15–1.14)	20.95–1.72 (1.74–1.72)	20.95–1.72 (1.74–1.72)	32.06–1.72 (1.73–1.72)	21.00–1.72 (1.74–1.72)
*R*_work_	0.1678 (0.3071)	0.1865 (0.3215)	0.1632 (0.2639)	0.1569 (0.2475)	0.1912 (0.3194)
*R*_free_	0.1821 (0.3207)	0.2146 (0.3428)	0.2568 (0.3386)	0.1894 (0.3100)	0.2348 (0.3595)
Average *B* factors (Å^2^)					
Protein	24.63		20.37	20.06	
Ligands (TPQ/Cu^2+^)	23.33		18.21	17.72	
Waters	39.77		41.08	35.54	
R.m.s. deviations					
Bond lengths (Å)	0.028		0.006	0.010	
Bond angles (°)	1.026		1.141	1.265	
No. of solvent atoms	1063		1115 [719§]	960 [806§]	
No. of multiple conformers	55		21	4	
Ramachandran plot (%)					
Favoured regions	96.2		96.7	96.8	
Allowed regions	3.7		3.1	3.0	
Outliers	0.1		0.2	0.2	

† Data from Murakawa *et al.* (2020 ▸).

‡ The low values of completeness and multiplicity are due to the elimination of some reflection spots with an elliptical shape (presumably derived from a distorted portion of the crystal) in the integration process.

§ Number of water molecules detected at approximately the same location (within 1.0 Å) as those detected by X-ray/neutron analysis.

## References

[bb1] Adams, P. D., Mustyakimov, M., Afonine, P. V. & Langan, P. (2009). *Acta Cryst.* D**65**, 567–573. 10.1107/S0907444909011548 PMC2685734 19465771

[bb2] Afonine, P. V., Mustyakimov, M., Grosse-Kunstleve, R. W., Moriarty, N. W., Langan, P. & Adams, P. D. (2010). *Acta Cryst.* D**66**, 1153–1163. 10.1107/S0907444910026582 PMC2967420 21041930

[bb3] Blakeley, M. P., Ruiz, F., Cachau, R., Hazemann, I., Meilleur, F., Mitschler, A., Ginell, S., Afonine, P., Ventura, O. N., Cousido-Siah, A., Haertlein, M., Joachimiak, A., Myles, D. & Podjarny, A. (2008). *Proc. Natl Acad. Sci. USA*, **105**, 1844–1848. 10.1073/pnas.0711659105 PMC2538850 18250329

[bb4] Brünger, A. T., Adams, P. D., Clore, G. M., DeLano, W. L., Gros, P., Grosse-Kunstleve, R. W., Jiang, J.-S., Kuszewski, J., Nilges, M., Pannu, N. S., Read, R. J., Rice, L. M., Simonson, T. & Warren, G. L. (1998). *Acta Cryst.* D**54**, 905–921. 10.1107/s0907444998003254 9757107

[bb5] Chen, V. B., Arendall, W. B., Headd, J. J., Keedy, D. A., Immormino, R. M., Kapral, G. J., Murray, L. W., Richardson, J. S. & Richardson, D. C. (2010). *Acta Cryst.* D**66**, 12–21. 10.1107/S0907444909042073 PMC2803126 20057044

[bb6] Chiu, Y.-C., Okajima, T., Murakawa, T., Uchida, M., Taki, M., Hirota, S., Kim, M., Yamaguchi, H., Kawano, Y., Kamiya, N., Kuroda, S., Hayashi, H., Yamamoto, Y. & Tanizawa, K. (2006). *Biochemistry*, **45**, 4105–4120. 10.1021/bi052464l 16566584

[bb8] Emsley, P., Lohkamp, B., Scott, W. G. & Cowtan, K. (2010). *Acta Cryst.* D**66**, 486–501. 10.1107/S0907444910007493 PMC2852313 20383002

[bb28] Hirano, Y., Takeda, K. & Miki, K. (2016). *Nature*, **534**, 281–284. 10.1038/nature18001 27279229

[bb10] J-PARC (2018). *J-PARC Annual Report 2018*, pp. 148–150. https://j-parc.jp/researcher/MatLife/ja/publication/files/MLF-AR_2018.pdf.

[bb11] Kabsch, W. (2010). *Acta Cryst.* D**66**, 125–132. 10.1107/S0907444909047337 PMC2815665 20124692

[bb12] Kim, M., Okajima, T., Kishishita, S., Yoshimura, M., Kawamori, A., Tanizawa, K. & Yamaguchi, H. (2002). *Nat. Struct. Biol.* **9**, 591–596. 10.1038/nsb824 12134140

[bb13] Kishishita, S., Okajima, T., Kim, M., Yamaguchi, H., Hirota, S., Suzuki, S., Kuroda, S., Tanizawa, K. & Mure, M. (2003). *J. Am. Chem. Soc.* **125**, 1041–1055. 10.1021/ja017899k 12537504

[bb14] Klema, V. J. & Wilmot, C. M. (2012). *Int. J. Mol. Sci.* **13**, 5375–5405. 10.3390/ijms13055375 PMC3382800 22754303

[bb15] Klinman, J. P. & Mu, D. (1994). *Annu. Rev. Biochem.* **63**, 299–344. 10.1146/annurev.bi.63.070194.001503 7979241

[bb16] Kusaka, K., Hosoya, T., Yamada, T., Tomoyori, K., Ohhara, T., Katagiri, M., Kurihara, K., Tanaka, I. & Niimura, N. (2013). *J. Synchrotron Rad.* **20**, 994–998. 10.1107/S0909049513021845 PMC3795571 24121355

[bb17] Liebschner, D., Afonine, P. V., Baker, M. L., Bunkóczi, G., Chen, V. B., Croll, T. I., Hintze, B., Hung, L.-W., Jain, S., McCoy, A. J., Moriarty, N. W., Oeffner, R. D., Poon, B. K., Prisant, M. G., Read, R. J., Richardson, J. S., Richardson, D. C., Sammito, M. D., Sobolev, O. V., Stockwell, D. H., Terwilliger, T. C., Urzhumtsev, A. G., Videau, L. L., Williams, C. J. & Adams, P. D. (2019). *Acta Cryst.* D**75**, 861–877.

[bb18] Liebschner, D., Afonine, P. V., Moriarty, N. W., Poon, B. K., Sobolev, O. V., Terwilliger, T. C. & Adams, P. D. (2017). *Acta Cryst.* D**73**, 148–157. 10.1107/S2059798316018210 PMC5297918 28177311

[bb19] Liebschner, D., Afonine, P. V., Urzhumtsev, A. G. & Adams, P. D. (2020). *Methods Enzymol.* **634**, 177–199. 10.1016/bs.mie.2020.01.007 PMC7574815 32093832

[bb20] Matsuzaki, R., Fukui, T., Sato, H., Ozaki, Y. & Tanizawa, K. (1994). *FEBS Lett.* **351**, 360–364. 10.1016/0014-5793(94)00884-1 8082796

[bb21] McCoy, A. J., Grosse-Kunstleve, R. W., Adams, P. D., Winn, M. D., Storoni, L. C. & Read, R. J. (2007). *J. Appl. Cryst.* **40**, 658–674. 10.1107/S0021889807021206 PMC2483472 19461840

[bb22] McIntire, W. S. & Hartmann, C. (1993). *Principles and Applications of Quinoproteins*, edited by V. L. Davidson, pp. 97–171. New York: Marcel Dekker.

[bb24] Murakawa, T., Hayashi, H., Sunami, T., Kurihara, K., Tamada, T., Kuroki, R., Suzuki, M., Tanizawa, K. & Okajima, T. (2013). *Acta Cryst.* D**69**, 2483–2494. 10.1107/S0907444913023196 24311589

[bb25] Murakawa, T., Kurihara, K., Shoji, M., Shibazaki, C., Sunami, T., Tamada, T., Yano, N., Yamada, T., Kusaka, K., Suzuki, M., Shigeta, Y., Kuroki, R., Hayashi, H., Yano, T., Tanizawa, K., Adachi, M. & Okajima, T. (2020). *Proc. Natl Acad. Sci. USA*, **117**, 10818–10824. 10.1073/pnas.1922538117 PMC7245091 32371483

[bb26] Nakajima, K., Kawakita, Y., Itoh, S., Abe, J., Aizawa, K., Aoki, H., Endo, H., Fujita, M., Funakoshi, K., Gong, W., Harada, M., Harjo, S., Hattori, T., Hino, M., Honda, T., Hoshikawa, A., Ikeda, K., Ino, T., Ishigaki, T., Ishikawa, Y., Iwase, H., Kai, T., Kajimoto, R., Kamiyama, T., Kaneko, N., Kawana, D., Ohira-Kawamura, S., Kawasaki, T., Kimura, A., Kiyanagi, R., Kojima, K., Kusaka, K., Lee, S., Machida, S., Masuda, T., Mishima, K., Mitamura, K., Nakamura, M., Nakamura, S., Nakao, A., Oda, T., Ohhara, T., Ohishi, K., Ohshita, H., Oikawa, K., Otomo, T., Sano-Furukawa, A., Shibata, K., Shinohara, T., Soyama, K., Suzuki, J., Suzuya, K., Takahara, A., Takata, S., Takeda, M., Toh, Y., Torii, S., Torikai, N., Yamada, N. L., Yamada, T., Yamazaki, D., Yokoo, T., Yonemura, M. & Yoshizawa, H. (2017). *Quantum Beam Sci.* **1**, 9.

[bb27] Niimura, N. & Podiarny, A. (2011). *Neutron Protein Crystallography: Hydrogen, Protons, and Hydration in Bio-macromolecules*. Oxford University Press.

[bb9] Ogata, H., Nishikawa, K. & Lubitz, W. (2015). *Nature*, **520**, 571–574. 10.1038/nature14110 25624102

[bb29] Okajima, T. & Tanizawa, K. (2009). *Copper Amine Oxidases: Structures, Catalytic Mechanisms and Role in Pathophysiology*, edited by G. Floris & B. Mondovi, pp. 113–118. Boca Raton: CRC Press.

[bb30] Schröder, G. C. & Meilleur, F. (2020). *J. Vis. Exp.*, e61903. 10.3791/61903 33346193

[bb31] Shoji, M., Murakawa, T., Boero, M., Shigeta, Y., Hayashi, H. & Okajima, T. (2020). *RSC Adv.* **10**, 38631–38639. 10.1039/d0ra06365g PMC9057271 35517562

[bb32] Tanaka, I., Chatake, T., Fujiwara, S., Hosoya, T., Kusaka, K., Niimura, N., Yamada, T. & Yano, N. (2020). *Methods Enzymol.* **634**, 101–123. 10.1016/bs.mie.2020.01.002 32093829

[bb33] Unno, M., Ishikawa-Suto, K., Kusaka, K., Tamada, T., Hagiwara, Y., Sugishima, M., Wada, K., Yamada, T., Tomoyori, K., Hosoya, T., Tanaka, I., Niimura, N., Kuroki, R., Inaka, K., Ishihara, M. & Fukuyama, K. (2015). *J. Am. Chem. Soc.* **137**, 5452–5460. 10.1021/jacs.5b00645 25872660

[bb34] Wlodawer, A. & Hendrickson, W. A. (1982). *Acta Cryst.* A**38**, 239–247.

[bb35] Yamaguchi, S., Kamikubo, H., Kurihara, K., Kuroki, R., Niimura, N., Shimizu, N., Yamazaki, Y. & Kataoka, M. (2009). *Proc. Natl Acad. Sci. USA*, **106**, 440–444. 10.1073/pnas.0811882106 PMC2626721 19122140

[bb36] Yano, N., Yamada, T., Hosoya, T., Ohhara, T., Tanaka, I., Niimura, N. & Kusaka, K. (2018). *Acta Cryst.* D**74**, 1041–1052. 10.1107/S2059798318012081 PMC6213574 30387763

