# Supplementary material for: Re-evaluation of protein neutron crystallography with and without X-ray/neutron joint refinement
Source: IUCrJ. 2022 Apr 8;9(Pt 3):342–8. doi: 10.1107/S2052252522003657 (PMC9067118; doi:10.1107/S2052252522003657)
Supplement: Supplementary file 1 [file m-09-00342-sup1.pdf]

IUCrJ

Volume 9 (2022)

**Supporting information for article:**

# Re-evaluation of protein neutron crystallography with and without X-ray/neutron joint refinement

Takeshi Murakawa, Kazuo Kurihara, Motoyasu Adachi, Katsuhiro Kusaka, Katsuyuki Tanizawa and Toshihide Okajima

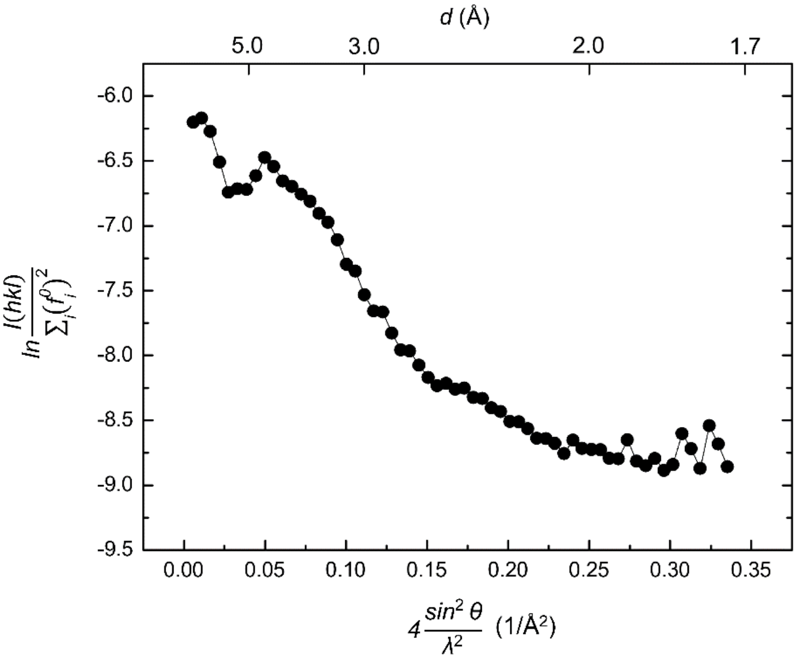


**Figure S1** Wilson plot of neutron diffraction data (Murakawa *et al*., 2020).

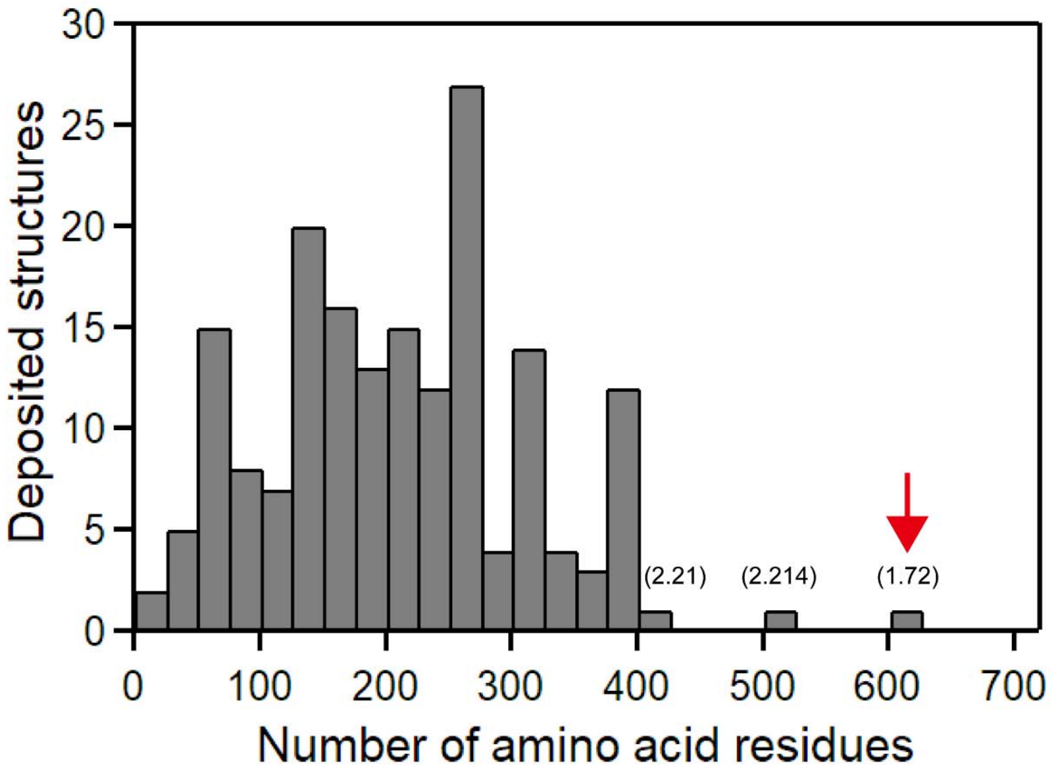


**Figure S2** Correlation between the number of amino acid residues in proteins and deposited structures determined by neutron crystallography. Resolution values are denoted in parentheses (in Å) for the structures with more than 400 amino acid residues. The red arrow indicates the data point for AGAO (Murakawa *et al*., 2020).

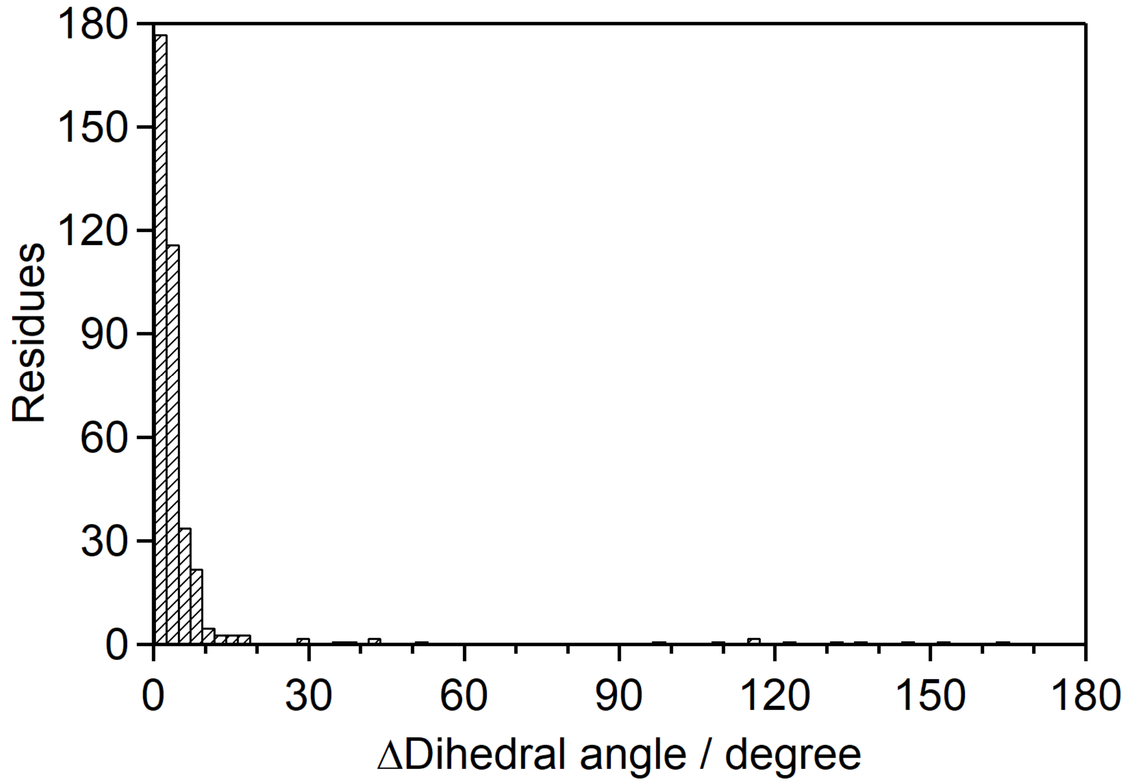


**Figure S3** Variance of side-chain orientations. Differences in the side-chain dihedral angle (N−Cα−Cβ−Cγ) between X-ray/neutron and neutron analyses are shown by the histogram.

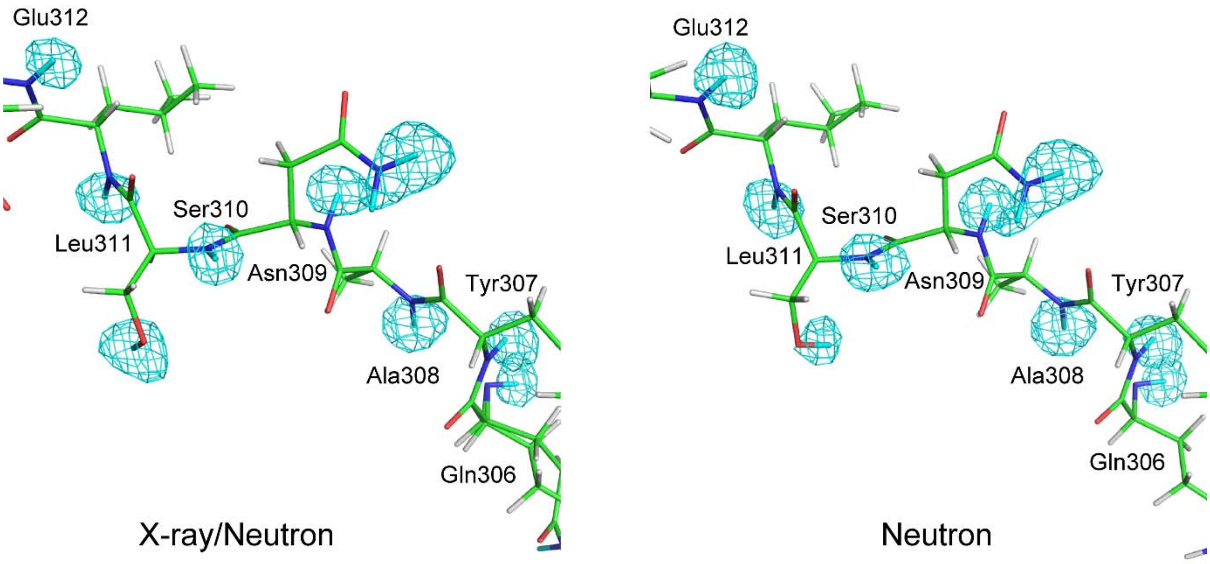


**Figure S4** $F_o - F_c$ polder omit SLD maps of deuterium atoms refined by X-ray/neutron analysis (left) and neutron analysis (right). The $F_o - F_c$ polder omit SLD maps between Tyr306 and Glu312 are presented. The cyan mesh is contoured at 4.0 σ. Note that only the Dγ atom of Ser310 was located at a different position.

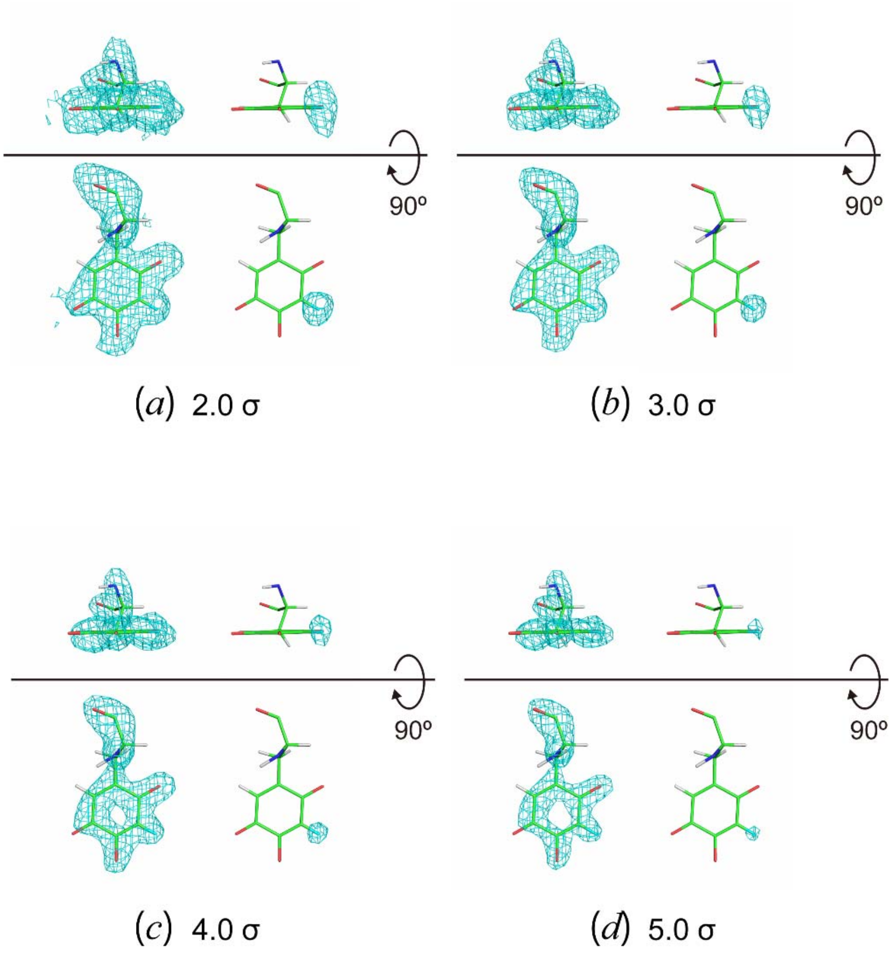


**Figure S5** Refined structure of TPQ from neutron analysis. The assigned model of TPQ is superimposed on the $F_o - F_c$ polder omit SLD map for residue 382 and deuterium atom attached to the C3 carbon of residue 382 contoured at 2.0 σ (a), 3.0 σ (b), 4.0 σ (c), and 5.0 σ (d), respectively.

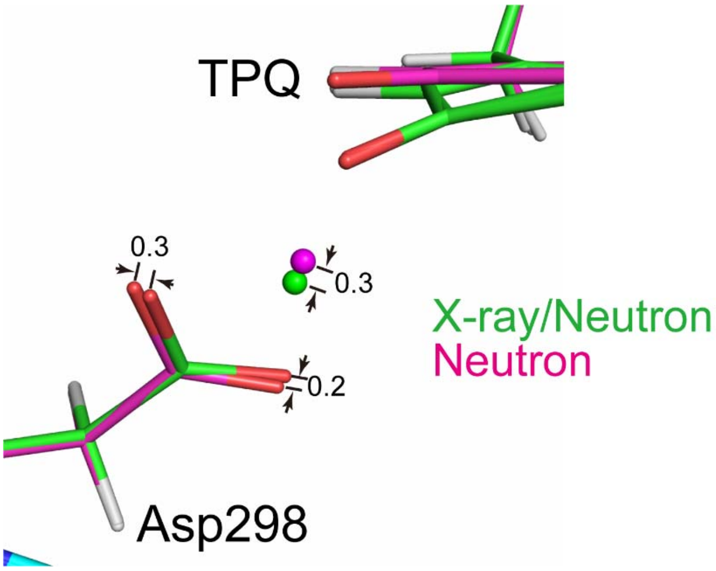


**Figure S6** Superimposed structure of triply shared proton between the cofactor and the catalytic base refined by X-ray/neutron analysis (green) and neutron analysis (magenta). Arrows represent the distances (Å).
